# Visfatin impact on the proteome of porcine luteal cells during implantation

**DOI:** 10.1038/s41598-024-65577-1

**Published:** 2024-06-25

**Authors:** Grzegorz Kopij, Marta Kiezun, Marlena Gudelska, Kamil Dobrzyn, Barbara Zarzecka, Edyta Rytelewska, Ewa Zaobidna, Bianka Swiderska, Agata Malinowska, Agnieszka Rak, Tadeusz Kaminski, Nina Smolinska

**Affiliations:** 1 Faculty of Biology and Biotechnology, University of Warmia and Mazury in Olsztyn, Olsztyn, Poland; 2 School of Medicine, Collegium Medicum, University of Warmia and Mazury in Olsztyn, Olsztyn, Poland; 3 Mass Spectrometry Laboratory, Institute of Biochemistry and Biophysics PAS in Warsaw, Warsaw, Poland; 4 Institute of Zoology and Biomedical Research, Jagiellonian University in Krakow, Kraków, Poland

**Keywords:** Visfatin, Pig, Corpora lutea, Luteal cells, Proteome, Implantation, Early pregnancy, LC–MS, Proteomics, Reproductive biology

## Abstract

Visfatin (VIS) is a hormone belonging to the adipokines’ group secreted mainly by the adipose tissue. VIS plays a crucial role in the control of energy homeostasis, inflammation, cell differentiation, and angiogenesis. VIS expression was confirmed in the hypothalamic–pituitary–gonadal (HPG) axis structures, as well as in the uterus, placenta, and conceptuses. We hypothesised that VIS may affect the abundance of proteins involved in the regulation of key processes occurring in the corpus luteum (CL) during the implantation process in pigs. In the present study, we performed the high-throughput proteomic analysis (liquid chromatography with tandem mass spectrometry, LC–MS/MS) to examine the in vitro influence of VIS (100 ng/mL) on differentially regulated proteins (DRPs) in the porcine luteal cells (LCs) on days 15–16 of pregnancy (implantation period). We have identified 511 DRPs, 276 of them were up-regulated, and 235 down-regulated in the presence of VIS. Revealed DRPs were assigned to 162 gene ontology terms. Western blot analysis of five chosen DRPs, ADAM metallopeptidase with thrombospondin type 1 motif 1 (ADAMTS1), lanosterol 14-α demethylase (CYP51A1), inhibin subunit beta A (INHBA), notch receptor 3 (NOTCH3), and prostaglandin E synthase 2 (mPGES2) confirmed the veracity and accuracy of LC–MS/MS method. We indicated that VIS modulates the expression of proteins connected with the regulation of lipogenesis and cholesterologenesis, and, in consequence, may be involved in the synthesis of steroid hormones, as well as prostaglandins’ metabolism. Moreover, we revealed that VIS affects the abundance of protein associated with ovarian cell proliferation, differentiation, and apoptosis, as well as CL new vessel formation and tissue remodelling. Our results suggest important roles for VIS in the regulation of ovarian functions during the peri-implantation period.

## Introduction

The corpus luteum (CL) is a temporary endocrine gland that regulates the course of the oestrous cycle and pregnancy. Across the oestrous cycle, the lifespan of CL can be divided into three phases: the formation: differentiation and luteinization of theca (Thc) and granulosa cells (Gc) to luteal cells (LCs), maintenance: inhibition of the luteolytic effect of prostaglandin F_2α_ (PGF_2α_) and release of progesterone (P_4_), and regression: reduction of steroidogenic enzyme activity and P_4_ production induced by the pulsatile endometrial secretion of PGF_2α_. During the early phase of gestation, the presence of embryos migrating within the uterus is signalled to the mother in a series of processes known as maternal recognition of pregnancy. In pigs, this period falls on days 10–13 of pregnancy. During this time, molecular signals such as growth factors are exchanged between the embryos, uterus and ovaries. Oestradiol (E_2_), synthesized and secreted by the embryos, exerts luteoprotective properties through the reduction of PGF_2α_ secretion and stimulation of luteinising hormone receptors’ (LHR) expression in CL. Furthermore, E_2_ increases the secretion of P_4_ and PGE_2_, stimulating the development of CL. On days 15–30 of porcine gestation, the observed second peak of embryo-derived E_2_ secretion ensures fully functional CL maintenance through undisrupted P_4_ and PGE_2_ synthesis and secretion. The proper course of both events is crucial for the preparation of ovaries and uterus, in the case of successful fertilization, to the proper course of implantation (15–16 days) and placentation (30–32 days). On days 15–16, the conceptuses begin attachment to the uterine endometrium. This is an essential stage because it marks the establishment of a stable connection between the conceptus and the maternal uterine tissue, which is necessary for successful pregnancy progression. In this period, the conceptuses undergo rapid growth and begin to develop structures necessary for nutrient exchange with the maternal blood supply, and development of the placenta and ensure proper nourishment and support for the growing embryos. As was indicated, between days 12 to 30 of pregnancy observed the highest mortality of embryos in a results of insufficient communication between the embryo and the mother’s organism^1^. The pregnancy may be lost through, among others, an increase in PGF_2_ secretion, CL luteolysis and, in consequence, inhibition of P_4_ production. PGF_2α_, whose highest secretion level is observed in the late luteal phase of the cycle, causes structural luteolysis and degeneration of luteal tissue in case the fertilisation or implantation does not occur. The elevated concentration of this prostaglandin during pregnancy may result in loss of labor^2–6^. Disruption of CL functioning may be the reason for many pathologies leading to the functional impairment of ovaries at both, structural and hormonal levels. CL insufficiency, irregularities of the luteal phase length, as well as abnormalities of steroidogenesis and endocrine hormone release, may lead to reproductive disorders, early embryonic loss, pregnancy failure, or even infertility. A number of CL-derived factors may play a crucial role not only in the regulation of ovarian functions but also in the modulation of uterine physiology. The disorders of CL functioning may affect the uterine functioning leading to embryo–endometrial asynchrony, disruption of embryo implantation, and failure to maintain pregnancy^2,3,5,6^.

Visfatin (VIS) is a 52 kDa protein belonging to the group of hormones secreted mainly by the white adipose tissue, called adipokines^7^. A specific receptor of VIS has not been identified; however, it is suggested that the adipokine may exert its influence by the phosphorylation of the insulin receptor. What is important, VIS does not compete with insulin for the receptor, which suggests that the adipokine binds to another site of the receptor protein^7^. VIS was found to exert pleiotropic actions in the organism, including the regulation of the inflammatory response^8–10^, energy homeostasis, insulin-mimetic activity^10,11^, as well as antiapoptotic actions^9,12^, cell differentiation^10^, and angiogenesis^13–15^. Increased levels of circulating VIS were correlated, among others, with tumorigenesis^16^.

In the ovaries, the expression of VIS was confirmed in the human Gc, cumulus cells and oocytes, as well as in the mouse and bovine Thc, Gc, cumulus cells, oocyte, stromal, and endothelial cells^17–19^. Our previous studies indicated also the expression of VIS in ovarian follicles of prepubertal and mature gilts as well as in porcine CL during the oestrous cycle and pregnancy^20,21^. Moreover, it was indicated that VIS is involved in the regulation of reproductive processes such as oocyte growth, its development and, maturation as well as ovulation. The adipokine has also been found to regulate steroid and prostaglandin secretion (for a review, see^17,18,22^). VIS was found to improve the developmental competency of oocyte and fertility potential in mice^17^. The adipokine also increased the cell proliferation rate, as well as E_2_ and P_4_ synthesis by the human Gc^19^. A similar effect of VIS was reported in cows and mice^17,18^. Ognjanovic and Bryant-Greenwood^8^ indicated the expression of VIS in the human foetal membranes^23,24^. Increased levels of VIS in the maternal serum were found to correlate with the human gestation progress^24,25^. In pregnant pigs, the highest levels of adipokine in blood plasma were observed on days 12–13, which relates to the maternal recognition of pregnancy in these animals^10,26^. The above data suggest that VIS may act as a crucial factor responsible for the maintenance of pregnancy. However, due to scarce literature data and a limited number of studies, the knowledge about the mechanisms of VIS actions in the female reproductive system requires further studies.

The high-throughput methods of analysis are highly effective tools in research concerning the role of individual factors in the physiology of cells, tissues, and organs. Transcriptomic and proteomic analyses reveal various biological processes occurring under specific conditions. Our previous research indicated the modulatory effect of VIS on the transcriptomic profile of the porcine LCs during the peri-implantation period using the high-throughput transcriptome sequencing (RNA-Seq)^27^. However, because the gene expression cannot be directly related to the biological effects, the overall landscape of VIS impact on LCs needs to be completed by the data concerning the adipokine effect on the global protein expression as well. Therefore, we decided to verify the hypothesis assuming that VIS may influence the proteome of porcine LCs during implantation and thus affect the production of many factors necessary for the maintenance and proper course of pregnancy. To our knowledge, this is the first study presenting the impact of VIS on the porcine proteomic profile of LCs during the implantation period (days 15 to 16 of gestation) with the use of liquid chromatography connected with tandem mass spectrometry (LC–MS/MS).

## Materials and methods

### Collection of samples

All experiments and methods were performed in accordance with relevant guidelines and regulations. The study was performed on four female cross-bred mature gilts (7–8 months of age and 130–150 kg in weight, n = 4) on days 15–16 of pregnancy, obtained from the breeding farm in Bałcyny, belonging to the University of Warmia and Mazury in Olsztyn, Poland. Gilts were daily verified for oestrous behaviour in the presence of a boar. Sows were naturally mated on days 1 to 2 of the oestrous cycle. The next day after coitus was denoted as the first day of pregnancy. Ovaries were collected immediately after slaughter, placed in cold phosphate-buffered saline (PBS) with the addition of 100 IU/mL penicillin and 100 µg/mL streptomycin, and transported to the laboratory for further in vitro procedure. The presence, size, and morphology of conceptuses collected from uterine horns^28^ as well as ovarian morphology^29^ were used to confirm the day of pregnancy. Tissue samples were harvested from animals intended for commercial slaughter and meat processing, and the collected tissues were an abattoir by-product. Sows were stunned by electricity (electronarcosis) and bled to death by cutting the carotid artery (exsanguination) in the commercial slaughter, according to European legislation (EFSA, AHAW/04-027). According to the Polish Act of 15 January 2015 (Journal of Laws, 2015, item 266) and the European Parliament Act of 22 September 2010 (Directive 2010/63/EU) on the protection of animals used for scientific or educational purposes, the experiments did not require the consent of the competent ethics committee for animal experiments. The study was designed in accordance with ARRIVE guidelines.

### Cell cultures

The isolation and in vitro culture procedure of LCs was performed according to Rytelewska et al.^30^. Luteal cells isolated from each animal (n = 4) were divided into two groups: the VIS-treated and the control group. Cells were sowed on the four 6-wells plates, two for further LC–MS and two Western Blot analysis. Cells were preincubated for 48 h in a humidified incubator (37 °C; 95% air, 5% CO_2_). After preincubation media were replaced and the cells were treated with recombinant human VIS (Cat. No. 8424-VF, RD System, MN, Minneapolis, USA) at the concentration of 100 ng/mL or, in the case of the control group, without any treatment for the next 24 h. Each n represents LCs originating from the same animal. The dose of VIS was determined based on Reverchon et al.^18^ and our previous study^27^. The viability of the cells and toxicity of the treatment were analysed using the AlamarBlue™ (Thermo Fisher Scientific, MA, Waltham, USA) assay according to the manufacturer’s instructions. The average reduction of AlamarBlue™ in the cultured LCs was 107.93 ± 2.91% in the VIS-treated group compared to the control group and confirmed that the treatment did not affect the viability of the cultured cells.

### Protein isolation

The protein isolation was performed using cell lysis buffer containing 30 mM Tris–HCl (pH 8), 8 M urea, and 2% CHAPS. After discarding the media, cells were treated with lysis buffer (100–150 µL per well) and incubated for 30 min on ice. Subsequently, the lysates were collected and mixed on a vortex three times for 15 s. In the next step, the samples were placed at 4 °C, incubated for 45 min, and mixed every 15 min as described above. Finally, the samples were centrifuged (10,000×*g* for 5 min at 4 °C) and supernatants were moved into the new, sterile tubes. Estimation of the total protein concentrations in samples was performed with the use of the Bradford procedure. Samples were stored at − 80 °C for further analysis. Protein isolation, and further protein digestion, and MS analysis was performed individually for all samples used in this experiment.

### Protein digestion

Proteomic analysis was performed at the Mass Spectrometry Laboratory at the Institute of Biochemistry and Biophysics of the Polish Academy of Sciences. Samples were digested using the filter-aided sample preparation (FASP) method^31^ according to the previously described protocol^32^, with minor modifications. In brief, 150 µg of total proteins was incubated for an hour with 20 mM tris(2-carboxyethyl)phosphine (TCEP) at 60 °C to reduce cysteine residues. Samples were transferred onto Vivacon 30 kDa molecular weight cut-off filter (Sartorius Stedim Biotech, Göttingen, Germany), spun at 14,000×*g* for 20 min, and washed with 100 µL urea solution (8 M urea in 100 mM triethylammonium bicarbonate buffer (TEAB)). Next, they were incubated for 10 min with 50 mM s-methylmethanethiosulfonate (MMTS) at room temperature. Samples were washed three times with 8 M urea buffer and 100 mM TEAB. Digestion was carried out overnight using 5 µg of trypsin (Promega GmbH, Mannheim, Germany) at 37 °C. Peptides were eluted from spin filters by two washes with 100 mM TEAB and one wash with 500 mM NaCl solution. Peptide concentrations were measured with Pierce™ Quantitative Colorimetric Peptide Assay (Thermo Fisher Scientific). 30 μg of peptides from each sample were labelled with TMTpro 16plex (Thermo Fisher Scientific) tags in 50 µL acetonitrile (ACN) for 1 h on vortex. The first tag was used to label an internal standard (IS), which was prepared by mixing equal protein amounts from all samples. The reaction was quenched by the addition of 8 µL 5% hydroxylamine. After checking the labelling efficiency (above 99.5% for both TMT sets), peptides were combined and desalted using four 30 mg Oasis HLB columns (Waters, MA, USA). Briefly, cartridges were preconditioned with 1 mL methanol and washed with 1 mL 1.5% ACN and 0.1% formic acid (FA). Subsequently, samples were loaded and rinsed with 1 mL 1.5% ACN and 0.1% FA. Peptides were eluted from columns with 400 µL 90% ACN and 0.1% FA. Aliquots were combined, dried in SpeedVac, and resuspended in 500 µL of 2% ACN in 10 mM ammonium hydroxide (AH).

### Reversed-phase peptide fractionation at high pH

Tandem mass tag (TMT)-labelled peptides were fractionated using high-pH reverse-phase chromatography on an XBridge Peptide BEH C18 column (4.6 × 250 mm, 130 Å, 5 µm, Waters). Separation was performed at 0.8 mL/min flow rate for 100 min on Waters Acquity UPLC H-class system. Mobile phases consisted of water (A), ACN (B), and 100 mM AH solution (C). The percentage of phase C was kept at a constant 10% through the entire gradient. Fractions were collected every 1 min starting from the fourth minute of the run. The following gradient was applied: 5 to 8% solvent B for 3 min, 8 to 15% for 17 min, 15 to 25% for 35 min, 25 to 33% for 15 min, 33 to 50% for 16 min, 50 to 90% for 8 min, 4 min isocratic hold at 90% and the final column equilibration at 3% phase B for 2 min. The peptide elution profile was monitored at 214 nm by the ultraviolet detector. Fractions were dried in Speedvac and reconstituted in 50 µL Evosep solvent A (0.1% FA in water) by 30 min vortexing and 30 min sonication.

### Mass spectrometry

The obtained fractions were analysed using LC–MS/MS system composed of Evosep One (Evosep Biosystems, Odense, Denmark) directly coupled to Orbitrap Exploris 480 mass spectrometer (Thermo Fisher Scientific, Bremen, Germany). 20 µL of each fraction was loaded onto disposable Evotips C18 trap columns (Evosep Biosystems) as previously described^33^. Chromatography was conducted at the flow rate of 500 nL/min using 44 min (30 samples per day) preformed gradient on EV1106 analytical column (Dr Maisch C18 AQ, 1.9 µm beads, 150 µm ID, 15 cm long, Evosep Biosystems). Data was acquired in positive mode with a data-dependent method. MS1 resolution was set at 60,000 with the normalized AGC target of 300%, auto maximum inject time, and a scan range of 300 to 1700 m/z (mass-to-charge ratio). For MS2, resolution was set at 30,000 with the standard normalized AGC target, auto maximum inject time, and top 25 precursors within an isolation window of 1.2 m/z considered for MS/MS analysis. Dynamic exclusion was set at 20 s with the allowed mass tolerance of ± 10 ppm, precursor intensity threshold at 5^e3^, and precursor fit threshold at 70%. Precursors were fragmented with a normalized collision energy of 30%. TurboTMT resolution mode was set to ‘TMTpro Reagents’. The spray voltage was set to 2.1 kV, funnel RF level at 40, and heated capillary temperature at 275 °C.

### Data analysis

Data analysis was performed in the MaxQuant/Andromeda software suite (version 2.0.1.0)^34^ using the *Sus scorfa* full Uniprot database (version 2022_02). The search was conducted using the following parameters: enzyme—trypsin, fixed modification—Metylthio (C), variable modification—Oxidation (M). Reporter MS2 quantification was specified to obtain values for the quantitative analysis. TMTpro 16plex correction factors were set according to the manufacturer’s specifications. A reverse database was used for target/decoy statistical results validation with peptide and protein false discovery rate (FDR) set to 1%. Match between runs function was enabled. Data for protein groups was further processed in Perseus (version 1.6.15)^35^. Hits from the reversed database and proteins only identified by site and contaminants were removed. TMT reporter values were normalized on the average sum of intensity^36^. Reporter intensities were log_2_ transformed and proteins with less than 3 samples in at least one biological group were filtered out. Missing values were replaced with data from normal distribution (width 0.3, down shift 1.8). Hits from analysis samples reporter signals were then normalised using the internal reference scaling (IRS) method^36^. Subsequently, samples were assigned to the animals and normalized to remove the batch effect using the ComBat algorithm^37^. The significance threshold for the p-value resulting from a Student’s test was 0.05 and the fold-change cut-off was ± 1.1.

### Network and functional analysis

To analyse the involvement of differentially regulated proteins (DRPs) in the common biological processes, the enrichment ontology and pathway analyses were performed with the use of String software (version 11.5) with available frameworks: Gene Ontology (GO) and Kyoto Encyclopedia of Genes and Genomes (KEGG) databases^38–40^ (p-adjusted < 0.05).

### Validation of LC–MS/MS results by western blot

To verify the results of LC–MS/MS analysis, we have performed Western blot analysis for five chosen proteins: ADAM metallopeptidase with thrombospondin type 1 motif 1 (ADAMTS1), lanosterol 14-α demethylase (CYP51A1), inhibin subunit beta A (INHBA), notch receptor 3 (NOTCH3), prostaglandin E synthase 2 (mPGES2) with the expression altered under the influence of VIS. To obtain the total protein lysates, cells descended for Western blot analysis were isolated and homogenized on ice, with the use of T-PER Tissue Protein Extraction Reagent (Thermo Fisher Scientific) enriched with protease inhibitors (Sigma-Aldrich, MO, Saint Louis, USA), and cleared by centrifugation (10,000×*g*, 5 min, 4 °C). Western blot analyses were conducted according to Smolinska et al.^41^, with modifications. The TATA-binding protein (TBP; antibodies cat. number: LS-C358352; Lifespan Biosciences, WA, Lynnwood, USA; dilution: 1:5000) was used as the reference protein and loading control. 20 µg of total protein lysates were separated by SDS-PAGE electrophoresis in a 12.5% polyacrylamide gel for ADAMTS1, CYP51A1, INHBA, mPGES2, and 10% for NOTCH3 using Mini-PROTEAN^®^ Tetra Cell (Bio-Rad Laboratories Inc., CA, Hercules USA) and transferred onto the polyvinylidene fluoride membrane (PVDF; Roche Diagnostics, Mannheim, Germany). To avoid the non-specific bindings, membranes were blocked with 5% bovine serum albumin in TBST and then incubated overnight at 4 °C with the specific primary antibodies against: ADAMTS1 (1:250, Thermo Fisher Scientific, Invitrogen, cat. number: #720329); CYP51A1 (1:250, Thermo Fisher Scientific, Invitrogen, cat. number: #PA5-59516); INHBA (1:250; Thermo Fisher Scientific, Bioss, cat. number: #BS-1774R), NOTCH3 (1:250, Thermo Fisher Scientific, Invitrogen, cat. number: #PA5-89283), and PTGES2 (1:250; FineTest, Wuhan, China, cat. number: FNab06917). After washing, membranes were incubated with HRP-conjugated secondary antibodies (1.5 h, RT): goat anti-rabbit IgG (AP156P; Merck Millipore, Burlington, MA, USA) for ADAMTS1, CYP51A1, INHBA, NOTCH3, PTGES2 and TBP (1:5000). The visualization of immunocomplexes was carried out using chemiluminescence HRP substrate (Merck Millipore, MA, Burlington, USA) according to the manufacturer’s protocol, and visualized with an Azure 280 Imaging System (Azure Biosystems, Dublin, CA, USA). The results of the analysis were quantified by optical density (OD) analysis of immunocomplexes with Image Studio Lite v.5.2 (LI-COR, NE, Lincoln, USA). Data were presented as the ratio of examined protein relative to TBP protein in the arbitrary OD units. The normality of Western blot data distributions was confirmed using a Shapiro–Wilk test (p > 0.05), and the results were statistically checked by a Student’s T-test (p < 0.05) using Statistica software (Statsoft Inc., OK, Tulsa, USA). Data were presented as mean ± SEM (n = 4).

### Ethics approval and consent to participate

All experiments and methods were performed in accordance with relevant guidelines and regulations. Tissue samples were harvested from animals intended for commercial slaughter and meat processing, and the collected tissues were an abattoir by-product. Sows were euthanized in the slaughter according to European legislation (EFSA, AHAW/04-027). The study was conducted according to the Polish Act of the protection of animals used for educational or scientific purposes of 15 January 2015 and directive 2010/63/EU (Journal of Laws Dz.U. 2015 No. item 266) of the European Parliament of 22 September 2010 (2010/63/EU) on the protection of animals used for scientific objectives, so this study did not require the consent of the competent ethics committee for animal experiments. The study was designed in accordance with ARRIVE guidelines.

## Results

### Differentially regulated proteins and functional annotations (GO and KEGG)

**Figure 1:**
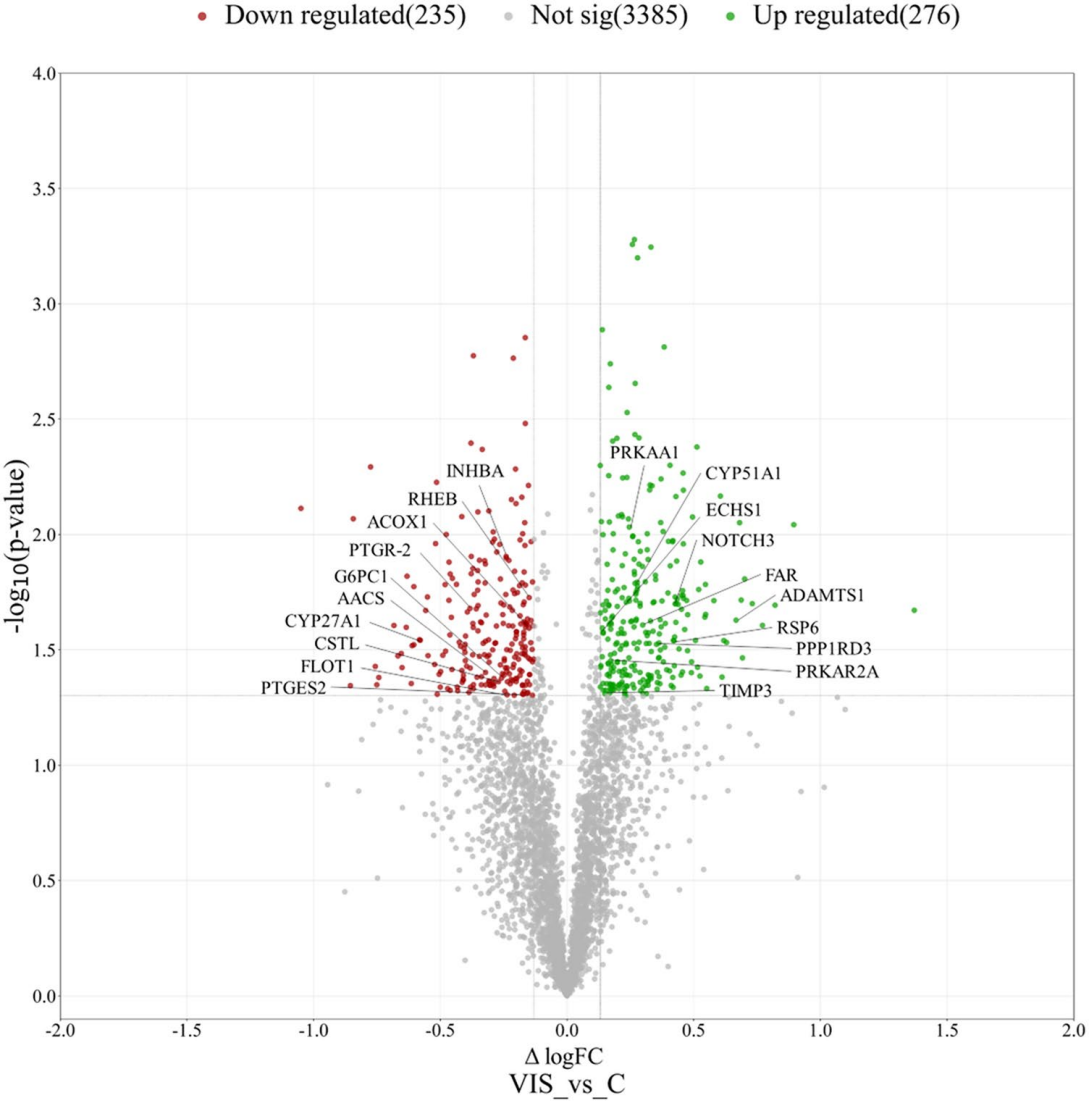
Volcano plot of the proteins identified by LC–MS/MS in VIS vs. C comparison. The volcano plot shows the fold-change (x-axis) versus the significance (y-axis) of the identified differentially regulated proteins (DRPs) under the influence of VIS. The significance (non-adjusted p-value) and the fold-change are converted to − log_10_(p-value) and log_2_(fold change), respectively. The vertical and horizontal dotted lines shows the cut-off of fold change =  ± 1.1 and of p-value < 0.05. The chosen DRPs are labeled by the protein symbols. *C* control group, *VIS* visfatin-treated group.

**Figure 2:**
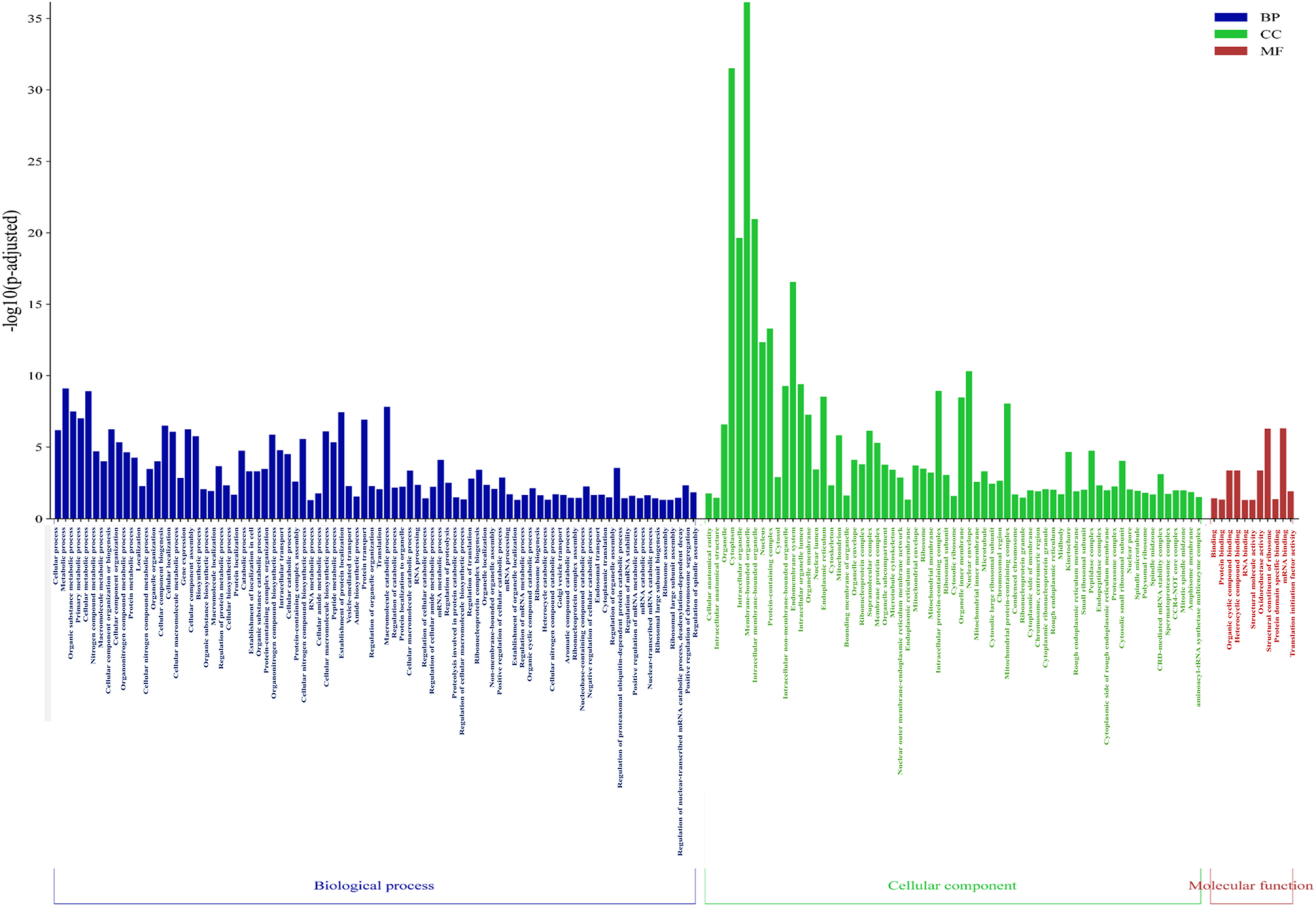
GO analysis of differentially regulated proteins (DRPs) in visfatin-treated group. Bar-plot illustrates the enrichment − log_10_(p-adjusted) analysis results. The functional terms are grouped and color-coded by data sources as follows: biological processes (BP) are blue, cellular components (CC) are green and molecular function (MF) are red. The x-axis represents GO terms, while the y-axis the enrichment score.

LC–MS/MS analysis revealed a total of 3896 proteins with expression changed under the influence of VIS (Supplementary Table 1). After the application of cut-off criteria (fold change cut-off ± 1.1, p < 0.05), we received the final list of 511 DRPs. DRPs were presented in the Volcano plot (Fig. 1). Among the identified DRPs, 276 were up-regulated and 235 were down-regulated in the presence of VIS (Supplementary Table 2). The fold change values ranged from − 2.07 (MSANTD3) to 2.59 (ANKRD17). The annotated DRPs were analysed using the String tool and classified into three GO categories: ‘biological processes’ (BP), ‘cellular components’ (CC), and ‘molecular function’ (MF) (Supplementary Table 3). DRPs were assigned to 162 GO terms (p-adjusted < 0.05); 86 of them were ascribed to BP, 65 to CC, and 11 to MF category (Fig. 2).

**Figure 3:**
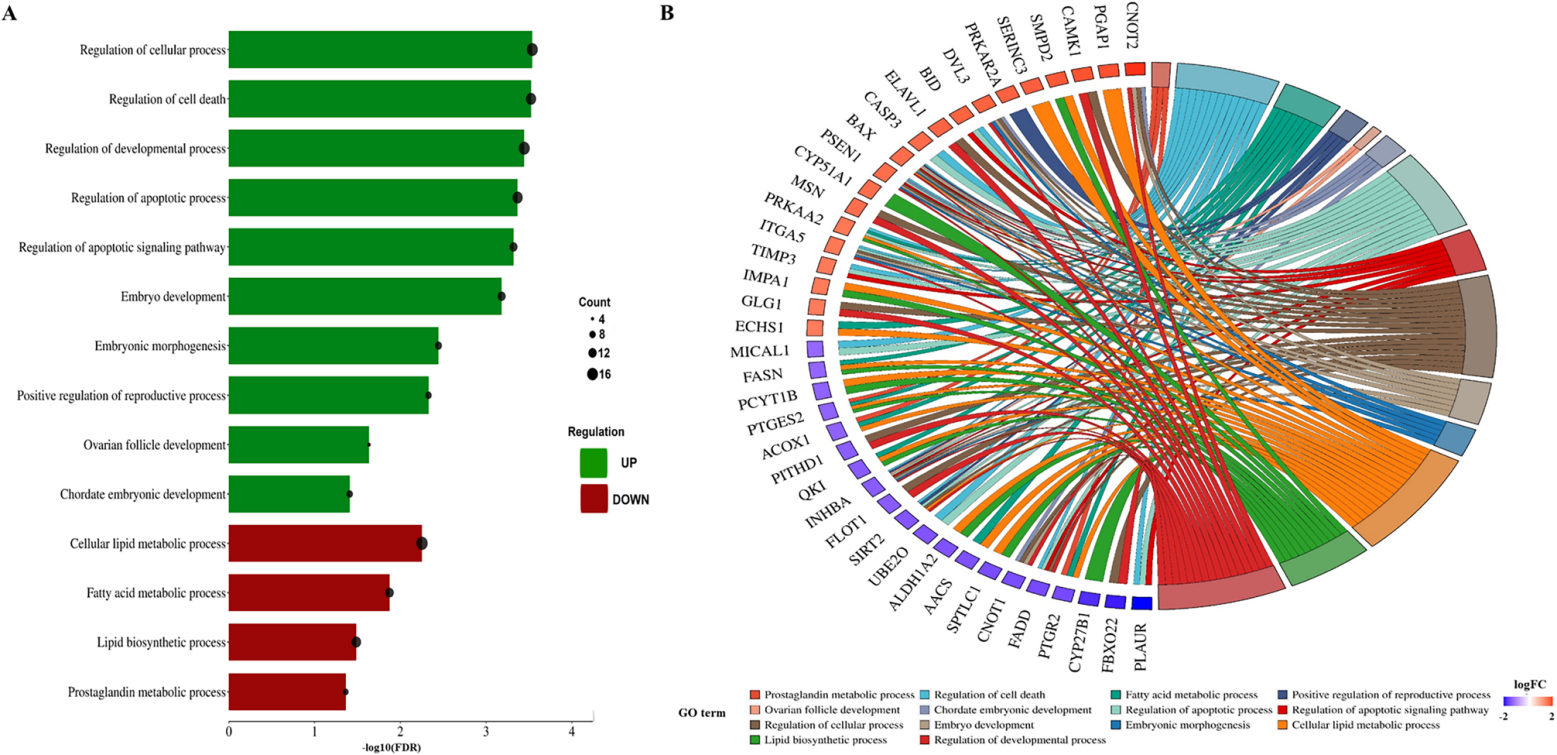
Visualization of the differentially regulated proteins (DRPs) determined to be statistically significant and their enrichment in the ontology terms detected by String tool evaluated in the luteal cells treated with visfatin. (**A**) The bar plot illustrates the fourteen most interesting GO biological processes enriched by DRPs. The colour of bars represents ratio between up- and downregulated proteins. Green bars show GO terms with the advantages of upregulated DRPs, whereas red bars represent terms consist of downregulated DRPs, mainly. The Y-axis describes GO terms and the X-axis shows − log_10_(FDR) for each enriched GO term. (**B**) The circular plot shows the selected GO terms enriched by DRPs.

In BP category, the highest number of DRPs were annotated to ‘cellular process’ (GO:0009987; 361 DRPs), ‘metabolic process’ (GO:0008152; 227 DRPs), ‘organic substance metabolic processes’ (GO:0071704; 210 DRPs), ‘primary metabolic process’ (GO:0044238; 200 DRPs), ‘cellular metabolic process’ (GO:0044237; 194 DRPs), ‘nitrogen compound metabolic process’ (GO:0006807; 178 DRPs), ‘macromolecule metabolic process’ (GO:0043170; 160 DRPs). From the most interesting processes enriched by the DRPs in BP subcategories we indicated: ‘lipid biosynthetic process’ (GO:0008610; 14 DRPs), ‘fatty acid metabolism process’ (GO:0006631; 11 DRPs), and ‘prostaglandin metabolic process’ (GO:0006693; 4 DRPs). We also found 14 DRPs ascribed to ‘regulation of apoptosis process’ (GO:0042981). Moreover, DRPs were annotated to ‘positive regulation of reproductive process’ (GO:2000243; 4 DRPs) and ‘ovarian follicle development’ (GO:0001541; 2 DRPs). Moreover, we found 19 DRPs annotated to ‘regulation of developmental process’ (GO:0050793; 19 DRPs). From which 7, 5, and 3 DRPs were ascribed to ‘embryo development’ (GO:0009790), ‘embryo morphogenesis’ (GO:0048598) and ‘chordate embryonic development’ (GO:0043009), respectively. The chosen enriched GO terms are shown in Fig. 3.

The highest number of DRPs in CC category were allocated to ‘cellular anatomical entity’ (GO:0110165; 409 DRPs), ‘intracellular anatomical organelle’ (GO:0005622; 376 DRPs), ‘cytoplasm organelle’ (GO:0043226; 332 DRPs), (GO:0005737; 330 DRPs), ‘membrane-bounded organelle’ (GO:0043227; 294 DRPs), and ‘intracellular membrane-bounded organelle’ (GO:0043231; 288 DRPs). The chosen enriched GO terms were shown in Fig. 2.

In MF category, the most enriched GO terms were ‘RNA biding’ (GO:0005488; 305 DRPs), ‘protein binding’ (GO:0005515; 176 DRPs), ‘organic cyclic compound binding’ (GO:0097159; 147 DRPs), and ‘heterocyclic compound biding’ (GO:1901363; 146 DRPs). The chosen enriched GO terms were shown in Fig. 2.

**Figure 4:**
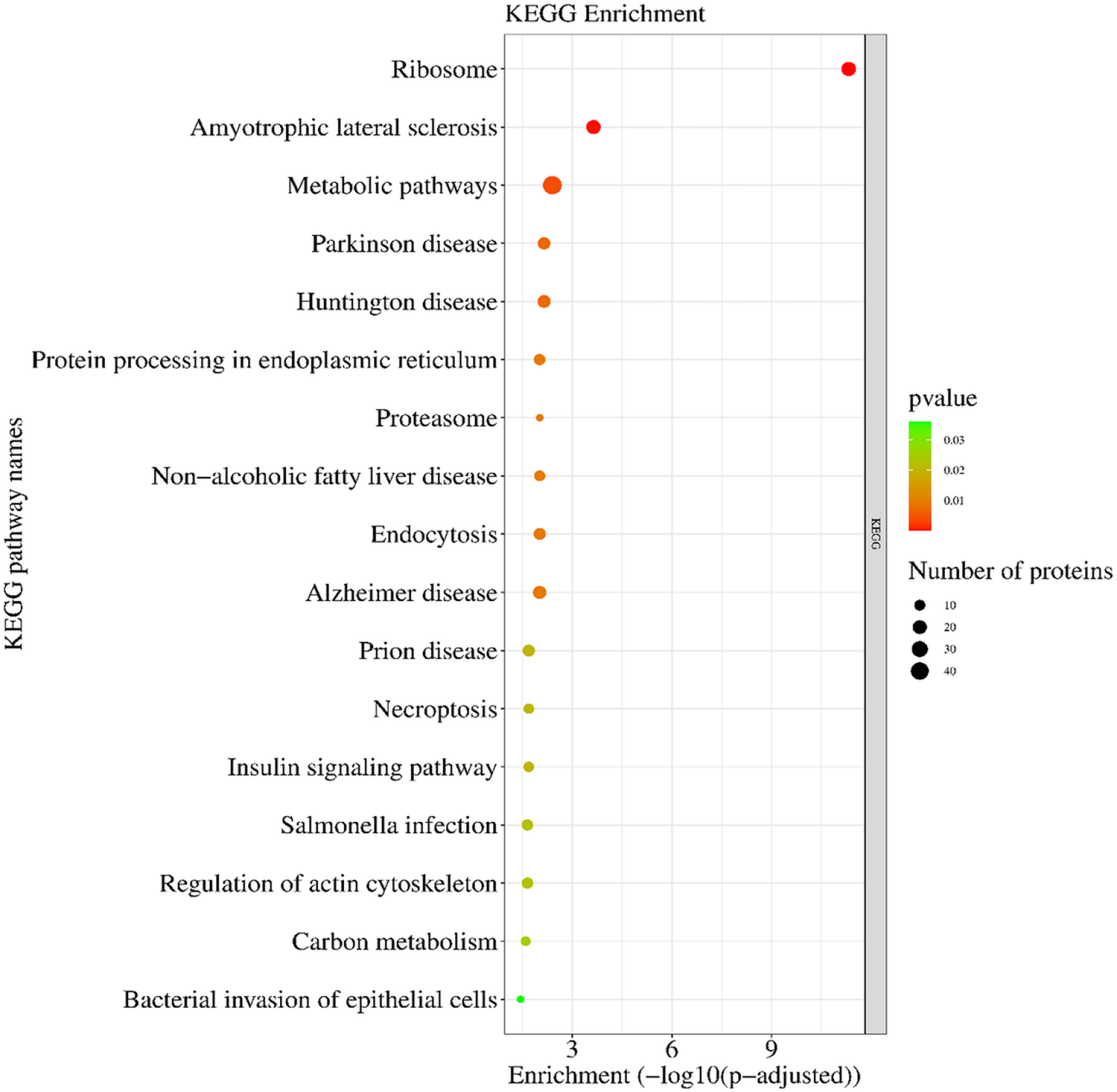
KEGG enrichment analysis differentially regulated proteins (DRPs) evaluated in the luteal cells treated with visfatin. Colour of dots represents DRPs statistically significant and their enrichment in the KEGG pathways. The size of dots, visualized the number of DRPs annotated to GO terms.

**Figure 5:**
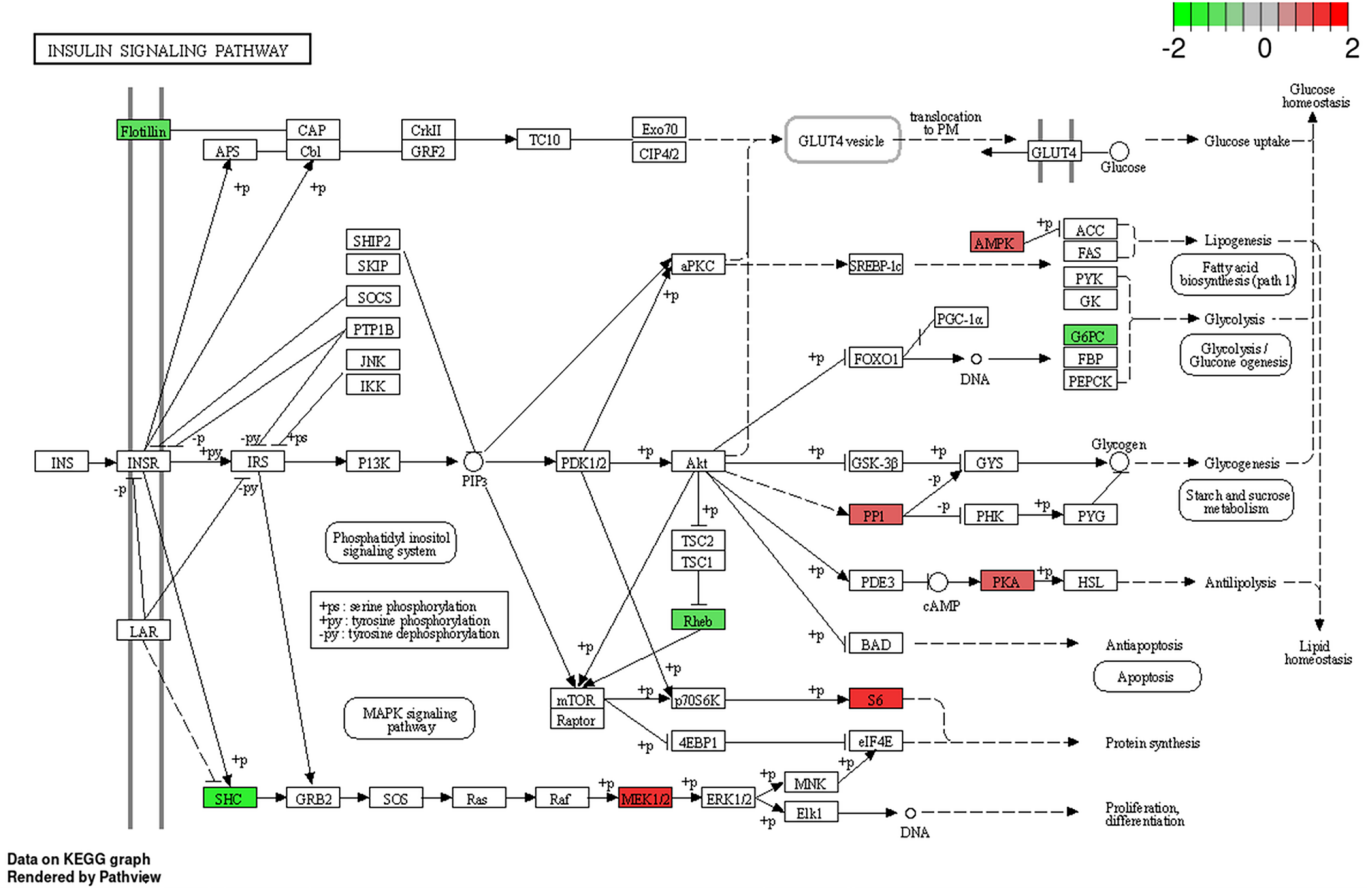
KEGG enrichment analysis differentially regulated proteins (DRPs) evaluated in the luteal cells treated with visfatin. The insulin signalling pathway enriched by nine differentially expressed proteins (DRPs). The red box shows upregulated genes and green box represents downregulated genes estimated based on log_2_(FC). Permission has been obtained from Kanehisa laboratories for using KEGG pathway database^40^.

KEGG enrichment analysis revealed proteins engagement in 17 pathways (Supplementary Table 3): ‘metabolic pathways’ (KEGG:ssc01100; 46 DRPs), ‘ribosome’ (KEGG:ssc03010; 22 DRPs), ‘amyotrophic lateral sclerosis’ (KEGG:ssc05014; 21 DRPs), ‘Alzheimer disease’ (KEGG:ssc05010; 17 DRPs), ‘Huntington disease’ (KEGG:ssc05016; 16 DRPs), ‘Parkinson disease’ (KEGG:ssc05012; 15 DRPs), ‘endocytosis’ (KEGG:ssc04144; 13 DRPs), ‘prion disease’ (KEGG:ssc05020; 13 DRPs), ‘salmonella infection’ (KEGG:ssc05132; 11 DRPs), ‘protein processing in endoplasmic reticulum’ (KEGG:ssc04141; 11 DRPs), ‘regulation of actin cytoskeleton’ (KEGG:ssc04810; 11 DRPs), ‘carbon metabolism’ (KEGG:ssc01200; 10 DRPs), ‘non-alcoholic fatty liver disease’ (KEGG:ssc04932; 10 DRPs), ‘necroptosis’ (KEGG:ssc04217; 9 DRPs), ‘insulin signalling pathway’ (KEGG:ssc04910; 9 DRPs), ‘proteasome’ (KEGG:ssc03050; 6 DRPs), and ‘bacterial invasion of epithelial cells’ (KEGG:ssc05100; 6 DRPs). The all results are shown in Figs. 4 and 5.

### Western blot

**Figure 6:**
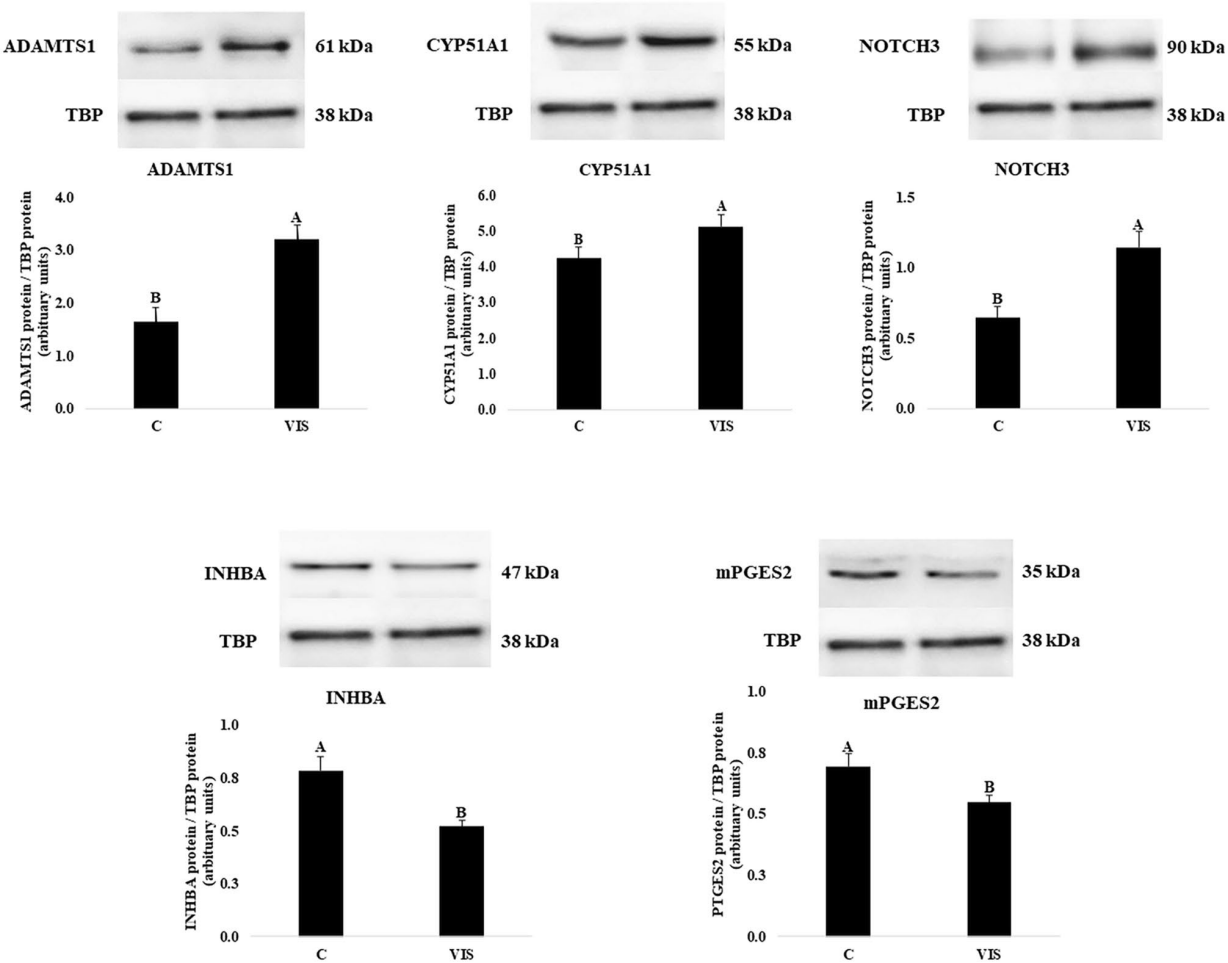
Western blot validation of LC–MS/MS results for differentially regulated proteins (DRPs) under visfatin influence in the porcine luteal cells. Validation was performed for ADAMTS1, CYP51A1, NOTCH3, INHBA and mPGES2 proteins with reference protein (TBP) (p-value < 0.05). Data are presented as the mean ± standard error of the mean (n = 4). Bars with different letters are significantly different at p < 0.05. The presented figures contain only the representative products for controls and VIS-treated groups. Original immunoblots are presented in the Supplementary File 1. *C* samples of the control groups, *VIS* samples of the VIS-treated groups, *ADAMTS1* ADAM metallopeptidase with thrombospondin type 1 motif 1, *CYP51A1* cytochrome P450 family 51 subfamily A member 1, *INHBA* inhibin subunit beta A, *NOTCH3* notch receptor 3, *mPGES2* prostaglandin E synthase 2, *TBP* TATA-binding protein (reference protein).

Validation of LC–MS/MS analysis was confirmed by Western blot technic for five chosen DRPs. In LCs of VIS-treated group compared to the control group the abundances of ADAMTS1, CYP51A1, and NOTCH3 were up-regulated (p < 0.05). The administration of VIS caused the down-regulation of INHBA and mPGES2 protein expression (p < 0.05). Western blot analyses agreed with those obtained during LC–MS/MS analysis and provided strong evidence for its reliability (Fig. 6).

## Discussion

To our knowledge, this in vitro study represents the first evidence of VIS impact on the proteome of porcine LCs during the implantation period. In this study, out of 511 statistically significant DRPs, 276 were up-regulated, and 235 were down-regulated under the influence of VIS. The identified DRPs were categorized into 162 GO functional annotations, some of which were associated with the regulation of apoptosis pathways. Additionally, they were annotated to the lipid and cholesterol metabolism, steroidogenesis as well as prostaglandin metabolic process. Some DRPs, through indirect mechanisms, were also linked to the embryo morphogenesis and development. We validated the results obtained through the high-throughput LC–MS/MS analysis using Western blot method, which provide strong evidence for the accuracy and reliability of the high-throughput method employed in this study.

VIS is a paracrine and autocrine signalling factor, influences various processes such as lipogenesis, lipid metabolism, and fatty acid synthesis. Additionally, the hormone promotes the storage of triglycerides in preadipocytes^7,42–44^. Our study seems to confirm the previous data as it revealed the stimulatory effect of VIS on the expression of acetyl-CoA carboxylase kinase (PRKAA2), fatty acyl-CoA reductase (FAR), enoyl-CoA hydratase (ECHS1), and lanosterol 14-alpha demethylase (CYP51A1), as well as the down-regulation of acetoacetyl-CoA synthetase (AACS), acyl-CoA oxidase 1 (ACOX1), cytochrome P450 family 27 subfamily B member 1 (CYP27A1), prostaglandin E synthase 2 (m-PGES2), prostaglandin reductase 2 (PTRG-2), and fatty acid synthase (FASN). The mentioned enzymes are engaged in the process of fatty acid synthesis, lipogenesis, and cholesterologenesis^45^. The proteins affected by VIS are associated with terms such as ‘lipid metabolic process’ (GO:006629), ‘cellular lipid metabolism process’ (GO:0044255), and ‘lipid biosynthetic process’ (GO:0008610), as indicated by GO analysis. Cholesterol, a vital bioactive sterol, modulates various signalling pathways and is crucial for follicular and CL development, as well as the functioning of gonads, embryonic development, and pregnancy maintenance. Cholesterol is the basic substrate of steroidogenesis processes in CL and can be converted into the steroid hormones, including P_4_, necessary to the maintenance of pregnancy^46,47^. In the case of embryos, cholesterol is obtained from the maternal organism^48^. Abnormalities of the synthesis and metabolism of cholesterol, as well as its transport and bile acids synthesis results in embryonic lethality and congenital defects^47,49^. Cholesterol can be synthesized de novo or absorbed from food^48^. De novo synthesis of fatty acids depends on the acetyl-CoA, acetyl-CoA carboxylases, and FASN complex^45^. Modulatory influence of VIS on the expression of precursor enzymes involved in lipogenesis and cholesterologenesis suggests that this adipokine may play a vital regulatory role in these processes.

VIS was found to regulate the steroidogenesis in the human mature ovaries^23,50^, influencing the synthesis of the steroidogenic acute regulatory protein (StAR) and 3β-hydroxysteroid dehydrogenase (3βHSD)^2,18,19^. VIS was also found to increase the expression of cytochrome P45019A1 (CYP19A1) and cytochrome P45011A1 (CYP11A1) in the bovine LCs^51^. During our data analysis, we observed the up-regulation of CYP51A1 and down-regulation of cytochrome P450 family 27 subfamily A member 1 (CYP27A1) expression in VIS-treated LCs. These proteins are crucial for different pathways of cholesterol degradation and are essential for sterols and steroids biosynthesis. CYP51A1 is a key regulator of converting lanosterol to cholesterol, which can further be transformed into steroids, as well as it is involved in the follicle-stimulating hormone (FSH)-regulated folliculogenesis and oocyte maturation^52^. The presence and high expression of CYP51A1 in LCs may affect steroidogenesis by the increased availability of cholesterol for P_4_ production, on which relies CL functioning. Conversely, dysregulation of CYP51A1 activity or expression could potentially impact P_4_ synthesis and compromise the maintenance of early pregnancy. The presence of CYP51A1 and farnesoid beta-receptor (FXRβ) in the pregnant female reproductive tract, including CLs, participate among others in fertilization, transport of gametes and zygotes as well as implantation^53^. In *CYP27A1* knock-out mice, urinary P_4_ concentrations were decreased, 20α-DH-progesterone increased, and the progesterone-to-20α-DH-progesterone ratio decreased three-fold. Thus, CYP27A1 modulates P_4_ concentrations. The authors proved that the underlying mechanism is the inhibition of 20α-hydroxysteroid dehydrogenase by 27-hydroxycholesterol, a product of CYP27A1^54^. CYP27A1 is an important protein for the progression of pregnancy and foetal organ development. CYP27A1-derived metabolites, such as oxysterols have been implicated in regulating immune responses and inflammation. During pregnancy, depletion of CYP27A1 was linked with higher concentrations of oxysterols and impaired differentiation and invasion of trophoblasts as well as lower litter size and higher mortality^55^. In our study, we indicated decreased expression of this protein in LCs treated by VIS, suggesting that the adipokine may play a role in regulating CYP27A1. Both CYP27A1 and CYP51A1 are part of the complex network of enzymes involved in cholesterol metabolism and steroidogenesis within the ovaries. Imbalances in cholesterol metabolism could lead to insufficient P_4_ production, which may result in pregnancy complications such as miscarriage or implantation failure. Moreover, NOTCH3, another protein identified in our study, was up-regulated in the VIS-treated LCs group. Recent research showed that the Notch pathway is active in both the embryonic and postnatal ovaries and is crucial for the follicle growth, synthesis and release of steroid hormones, and ovarian vascularization^56,57^. Notably, aberrations in Notch signalling have been linked to female reproductive disorders, including polycystic ovary syndrome (PCOS), follicle growth arrest, reduced Gc proliferation, and hyperplasia of Thc^56,57^. This suggests that Notch signalling is a novel and potent mechanism capable of regulating luteal steroidogenesis. Studies have suggested that Notch/PI3K/AKT signalling pathway may interact with P_4_ and enhance its anti-luteolytic properties in LCs^56,58^. Overexpression of the intracellular domain of NOTCH3 (NICD3) has been found to stimulate P_4_ secretion by regulating key enzymes involved in steroid biosynthesis, such as CYP11A1 and StAR^56^. Additionally, we observed that VIS stimulated tissue inhibitor of metalloproteinases 3 (TIMP3) protein expression, while inhibiting the abundance of cathepsin L (CTSL) protein. TIMP3 and CTSL contribute to stimulating steroidogenesis, particularly through their interaction with the phosphatidylinositol 3-kinase/Akt (PI3K/Akt) pathway^59–62^. This interaction has been associated with the synthesis of steroid hormones like P_4_ and E_2_, highlighting their potential role in regulating ovarian functions^61–63^. Our findings suggest that VIS, through its influence on the above mentioned proteins, may contribute to the synthesis of steroid hormones that are essential for various ovarian functions, including follicular development, ovulation, luteinization, and establishment and maintenance of pregnancy.

The obtained data also revealed the inhibitory influence of VIS on the expression of INHBA protein in the porcine LCs. This protein was ascribed to the ‘positive regulation of reproductive process’ (GO:2000243), ‘embryonic morphogenesis’ (GO:0048598), and ‘embryo development’ (GO:0009790), as well as ‘apoptotic process’ (GO:0006915). INHBA belongs to the transforming growth factor beta (TGFβ) superfamily and participates in the synthesis of βA-subunit of some activin and inhibin complexes^64^. Notably, in the gonadectomized rats, INHBA was undetectable in the blood plasma, suggesting that gonads are the primary source of this factor^65,66^. Inhibin and activin are peptide hormones secreted by various tissues, including Gc, Thc, CL, placenta, uteri, oocytes, and Sertoli cells^64–68^. The hormones are expressed by the cells derived from the sex cord-stromal lineage and play a crucial role in the control of FSH secretion by the pituitary gland^65–68^. Knockdown of INHBA led to a significant increase of FSHβ subunit expression, while overexpression of INHBA caused a decrease of its mRNA level^64^. Inhibin is recognized as a paracrine mediator involved in the regulation of ovarian folliculogenesis and steroidogenesis^64,66^. In the porcine Gc, the stimulation of INHBA led to increased secretion of E_2_ and cell proliferation and had a synergistic effect with FSH on E_2_ secretion^67^. Additionally, INHBA up-regulated genes encoding gonadotropin receptors, such as FSH receptor (FSHR) and luteinizing hormone receptor (LHR), which take part in steroidogenesis^67^. Notably, the expression of INHBA decreases under the influence of FSH but rises after the luteinizing hormone (LH) surge in the pig^67^. These findings suggest that VIS may be an important regulator of ovarian steroidogenesis, potentially also through its influence on INHBA production. It appears that VIS, through its effect on INHBA, may regulate the functioning of the hypothalamic–pituitary–gonadal (HPG) axis.

In our study, we also observed the inhibitory effect of VIS on mPGES2 and PTGR-2 protein abundance. These proteins are part of the overall lipid synthesis mechanism and are associated with the ‘prostaglandin metabolic process’ (GO:0006693). Prostaglandins (PGs) are central mediators of female reproductive processes, including ovulation, implantation, maintenance of pregnancy, and labour^69^. mPGES2, along with cyclooxygenases (COXs), is required to transform prostaglandin H_2_ (PGH_2_). During early pregnancy, mPGES2 may be involved in modulating the inflammatory and immune responses within the reproductive tract and the developing embryo. PGE_2_ produced by mPGES2 activity could contribute to processes such as embryo implantation, uterine remodelling, and immunomodulation to support the establishment and maintenance of pregnancy. Additionally, mPGES2-mediated PGE_2_ synthesis may interact with other pathways involved in early pregnancy, such as hormone signalling (e.g., P_4_) and vascular remodelling, to ensure proper development of the maternal-foetal interface and placenta^69,70^. PGE_2_ affects the maturation of oocytes and their development^69^. The hormone induces the expression of luteinizing hormone (LH) receptors in LCs, increasing P_4_ synthesis^69^. In our study, we also found the impact of VIS on PTGR-2 protein. PTGR-2 as reductase is involved in the metabolism and inactivation of PGs by the conversion of PGE to its less biologically active form^71^. The relationship between mPGES2 and PTGR-2 in CL during early pregnancy likely involves a delicate balance between prostaglandin synthesis and metabolism. mPGES2 may be upregulated to promote the synthesis of PGE_2_, which can have stimulatory effects on CL and P_4_ production. On the other hand, PTGR-2 may be involved in the metabolism of PGs, including PGE_2_, potentially regulating their levels and effects within CL. The interaction may prevent luteolysis and support the luteal function for embryo development and successful implantation is achieved through the increased PGE_2_:PGF_2α_ ratio during early pregnancy^69^. VIS, and its inhibition or silencing via siRNA, has demonstrated effects on PGE_2_ release and mPGES1 synthesis in mice chondrocytes^72^. In the mice models, VIS has been established as necessary for IL-1β-induced PGE_2_ synthesis. Considering that VIS modulates mPGES-2 and PTGR-2 proteins’ abundance, we can infer that VIS may be involved in the regulation of PGs synthesis pathways.

Ovarian transformation depends on extracellular matrix (ECM) remodelling and is tightly controlled by the specific factors, including proteases and their inhibitors such as TIMPs, ADAMTs, metalloproteinases (MMPs), and cathepsins^73–78^. In our study, we identified some ECM-related proteins, such as mentioned above TIMP3, ADAMTS1 that participate in ECM remodelling and tightly control this process. TIMP3 acts as a regulator of MMPs and ADAMs activity by binding to them and preventing from degrading ECM and protein turnover^61,62,76,77,79^. In mice, ADAMTS1, TIMP3 are the key factors of normal cell growth, organ morphology, fertility, CL formation, and shaping of ECM^59,62,73,75,76,80^. ADAMTS1 might have a crucial function in releasing ECM-bound factors, such as growth factors, that facilitate luteal development^81,82^. Reduced levels of TIMP3 during the luteal phase may allow for significant ADAMTS1 activity in this period. Despite the decrease of ADAMTS1 levels towards the end of the luteal phase, both mRNA and protein remained detectable, and any remaining ADAMTS1 activity could potentially be restrained by an increase of TIMP3 levels^76^. It seems the mutual relationship of these two proteins may be important during CL formation (ADAMTS1 predominates) and regression (TIMP3 predominates)^76^. Gosset et al.^72^ indicated the increased expression of several matrix proteases such as ADAM and MMPs in the human chondrocytes after stimulation with recombinant VIS. In our studies, VIS stimulated the expression of both ADAMTS1 (1.59) and TIMP3 (1.11) with a greater abundance of the first. Our observation seems to be consistent with the above assumption. It seems possible that the adipokine may act as a balancing factor between ADAMTS1/TIMP3 activity, indirectly taking part in the control of CL development and maintenance.

ECM remodelling is a process that plays a dual role, it triggers the cellular proliferation process and apoptosis. As was evidenced apoptosis acts as a signal to stimulate proliferation, angiogenesis as well as cell differentiation, and migration^83–85^. The identified proteins in our research, TIMP3, ADAMTS1and INHBA, have been associated by GO analysis with the regulation of apoptosis and proliferation processes. These processes are regulated by intricate pathways such as the extracellular signal-regulated kinase (ERK1/2), PI3K/Akt, and vascular endothelial growth factor/vascular endothelial growth factor receptor 2 (VEGF/VEGFR2) which can also be influenced by the aforementioned proteins^60,79,86–92^. Adya et al.^15^ highlighted the important role of VIS in the proliferation, apoptosis, angiogenesis, and capillary tube formation. VIS exerts stimulatory effects on VEGFR2 and VEGF expression, and it has anti-apoptotic effects by activating PI3K/Akt and ERK1/2 pathways. These effects were mediated through the regulation of MMPs and TIMPs^15^. TIMP3 can promote apoptosis by preventing cells or ECM components from shedding death receptors and their ligands. Increased TIMP3 in the regressing CL may influence programmed cell death of luteal tissue^93^. TIMP3 participates in VEGF pathway as an anti-angiogenic factor through the blockade of VEGFR2. This is crucial for controlling excessive blood vessel formation^88^. It may also play a role in preserving the established blood vessels in anticipation of dynamic alterations in angiogenesis that take place during the transition from the ovulatory follicle to the CL. This is because TIMP3 is involved in stabilizing the vascular endothelial cell tubes, preventing the regression of recently formed vascular networks^79,89,90,94^. Moreover, ADAMTS1 plays an important role in LCs survival, differentiation, and function during pregnancy^95^ by influencing PI3K/Akt and ERK1/2 pathways^87^. It is involved in modulating angiogenesis and vessel development in LCs, possibly through its thrombospondin motif, which may facilitate local angiogenesis by selectively binding with VEGF^91^. The collaboration of TIMP3, ADAMTS1, and CTSL controlled by VIS is essential for ensuring the proper function of LCs during pregnancy. Their interactions within the VEGF pathway, as well as their involvement in pathways like PI3K/Akt and ERK1/2, collectively contribute to the regulation of angiogenesis, vessel integrity, and the cellular environment.

Visfatin controls energy homeostasis as an insulin-mimetic protein by acting on the insulin signalling pathway^96^. In the mice pancreatic TC6 cell line (BTC), VIS increased the expression of some genes related to diabetes as well as *INS*. Moreover, VIS stimulated INS secretion as well as induces activation of the insulin receptor/ERK1/2 pathway, and using a blocker of this adipokine abolishes these effects^96^. VIS increased glucose uptake in the adipocytes and skeletal cells and decreased glucose release from the hepatocytes^23^. In our study, KEGG enrichment analysis revealed nine proteins, namely PRKAA, protein kinase 3ʹ,5ʹ-cyclic adenosine monophosphate (cAMP)-dependent type II regulatory subunit alpha (PRKAR2A), Ras homolog enriched in brain 2 (RHEB), ribosomal protein S6 (RPS6), mitogen-activated protein kinase 2 (MAP2K2), Src homology 2 domain-containing (SHC) transforming protein 1 (SHC1), protein phosphatase 1 regulatory subunit 3D (PPP1R3D), glucose-6-phosphatase catalytic subunit 2 (G6PC2), and flotillin 1 (FLOT1), which are engaged in the ‘insulin signalling pathway’ (Fig. 5). These proteins, including PRKAA2, PRKAR2A, RHEB, RPS6, and MAP2K2, play various roles in cellular processes such as metabolism, growth, and cell proliferation. For instance, PRKAA2 is involved in energy regulation and is associated with type 2 diabetes mellitus^97,98^. It plays a potential role in monitoring energy levels, which could be crucial for P_4_ synthesis in LCs. PRKAR2A’s involvement in the cellular responses to hormones may affect P_4_ production and the overall function of LCs^97,98^. RHEB, known for its role in the cell growth and mTOR signalling, could influence CL function and maintenance^99^. RPS6, essential for protein synthesis, might affect the enzymes and factors required for P_4_ production^99^. Similarly, the role of MAP2K2 in cell growth and differentiation may be relevant to LCs’ proliferation^99^. Our studies seem to confirm the involvement of VIS in the regulation of the insulin signalling pathway.

**Figure 7:**
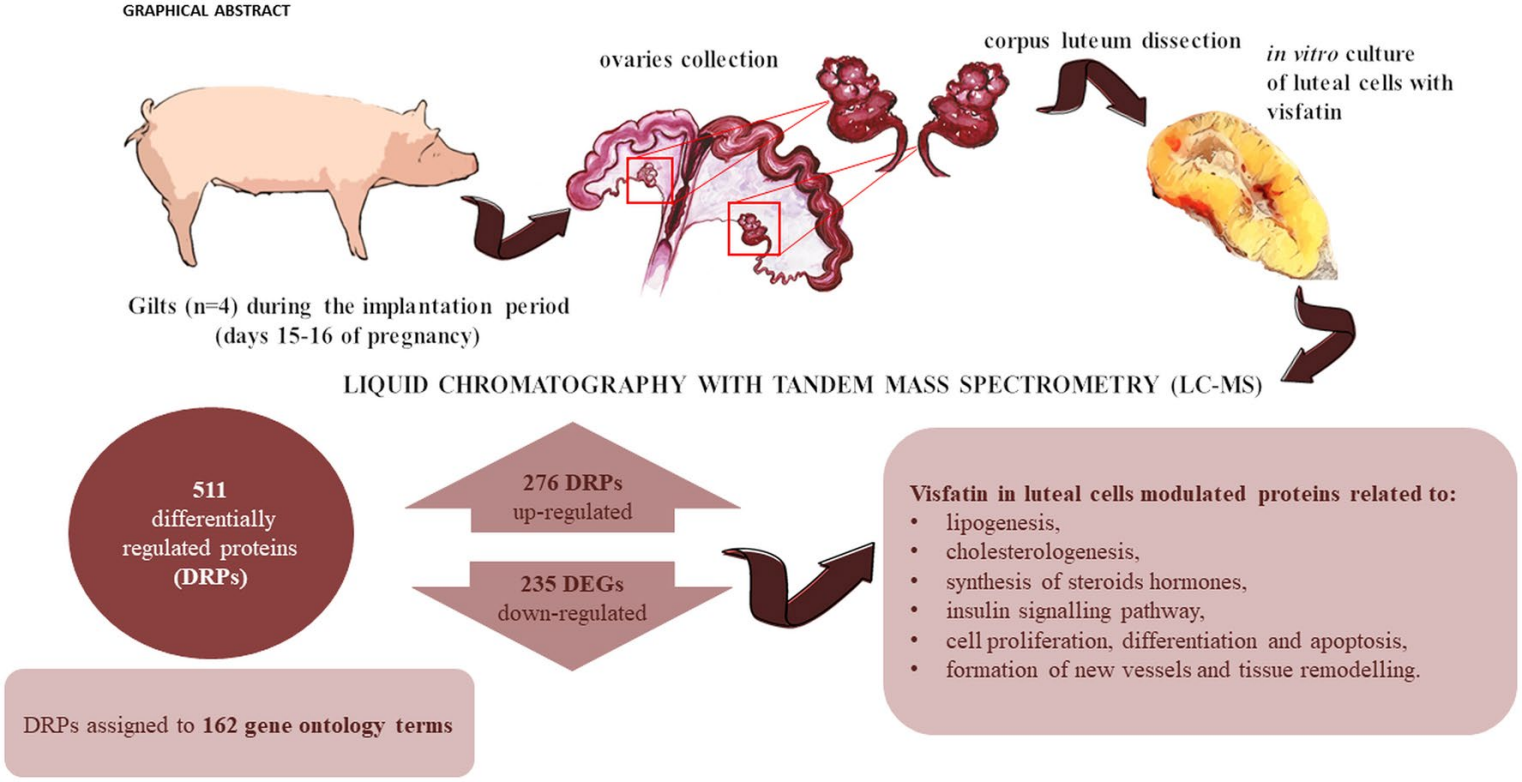
The graphical summary of the main findings of the study.

In this study, we have unveiled the influence of VIS on the protein concentrations of a multitude of proteins intricately tied to diverse processes. They encompass, among others, ovarian cell proliferation, differentiation, vascularization, angiogenesis, apoptosis, and ovarian development. Importantly, the impact of VIS on proteins associated with these processes implies its indirect modulation of ovarian functions. This connection implies a potential role for VIS in the regulation of lipogenesis and cholesterologenesis, cascading down to influence broader metabolic pathways. Furthermore, our investigation has shown another face of VIS impact: its ability to modulate the expression of proteins linked to the synthesis of steroid hormones. A notable discovery lies in VIS's effect on specific proteins related to insulin metabolism, a finding reinforced by KEGG enrichment analysis. The graphical summary of our findings has been presented in the Fig. 7. The intriguing association opens new avenues of exploration into the intricate interplay between VIS and metabolic pathways, with potential implications for understanding insulin-related disorders. Overall, our research lends substantial support to the hypothesis proposing a pivotal role for VIS in the orchestration of ovarian functions during the periimplantation period. As our results align with this hypothesis, they pave the way for deeper investigations into the molecular intricacies underpinning ovarian regulation and offer potential insights into therapeutic interventions targeting ovarian health.

### Supplementary Information

Supplementary Information.

Supplementary Legends.

Supplementary Table 1.

Supplementary Table 2.

Supplementary Table 3.

## Data Availability

The mass spectrometry proteomics data have been deposited to the ProteomeXchange Consortium via the PRIDE^100^ partner repository with the dataset identifier PXD047445 and 10.6019/PXD047445. Other data are available on request from the corresponding author.

## Abbreviations

3βHSD: 3β-Hydroxysteroid dehydrogenase
AACS: Acetoacetyl-CoA synthetase
ACN: Acetonitrile
ACOX1: Acyl-CoA oxidase 1
ADAMTS1: ADAM metallopeptidase with thrombospondin type 1 motif 1
BP: Biological processes
BTC: Mice pancreatic TC6 cell line
CC: Cellular components
CL: Corpus luteum
COX: Cyclooxygenase
CSTL: Cathepsin L
CYP11A1: Cytochrome P450 Family 11 Subfamily A Member 1
CYP19A1: Cytochrome P450 Family 19 Subfamily A Member 1
CYP27A1: Cytochrome P450 Family 27 Subfamily A Member 1
CYP51A1: Lanosterol 14-α demethylase
DRPs: Differentially regulated proteins
E_2_: Oestradiol
ECHS1: Enoyl-CoA hydratase
ECM: Extracellular matrix
ERK1/2: Extracellular signal-regulated kinase signalling pathway
FA: Formic acid
FAR: Fatty acyl-CoA reductase
FASN: Fatty acid synthase
FDR: False discovery rate
FLOT1: Flotillin 1
FSH: Follicle-stimulating hormone
FSHR: Follicle stimulating hormone receptor
G6PC2: Glucose-6-phosphatase catalytic subunit 2
Gc: Granulosa cells
GO: Gene Ontology
HOMECs: Human microvascular omental endothelial cells
HPG: Hypothalamic–pituitary–gonadal axis
HUVECs: Human umbilical vein endothelial cell
IL-1β: Interleukin 1 beta
INHBA: Inhibin subunit beta A
INS: Insulin
IRS: Internal reference scaling
IS: Internal standard
KEGG: Kyoto Encyclopedia of Genes and Genomes
LC-MS: Liquid chromatography with tandem mass spectrometry
LCs: Luteal cells
LH: Luteinizing hormone
LHR: Luteinizing hormone receptor
MAP2K2: Mitogen-activated protein kinase 2
MF: Molecular function
MMP: Metalloproteinase
MMTS: S-Methylmethanethiosulfonate
mPGES2: Prostaglandin E synthase 2
NICD3: Intracellular domain of NOTCH3
NOTCH3: Notch receptor 3
OD: Optical density
P_4_: Progesterone
PBS: Phosphate-buffered saline
PCOS: Polycystic ovary syndrome
PG: Prostaglandin
PGE_2_: Prostaglandin E_2_
PGF_2α_: Prostaglandin F_2α_
PGH_2_: Prostaglandin H_2_
PI3K/Akt: Phosphatidylinositol 3-kinase/Akt signalling pathway
PPP1R3D: Protein phosphatase 1 regulatory subunit 3D
PRKAA: Acetyl-CoA carboxylase kinase
PRKAR2A: Protein kinase 3ʹ,5ʹ-cyclic adenosine monophosphate (cAMP)-dependent type ii regulatory subunit alpha
PTRG-2: Prostaglandin reductase 2
RHEB: Ras homolog enriched in brain 2
RNA-Seq: High-throughput transcriptome sequencing
RSP6: Ribosomal protein S6
RT: Room temperature
SEM: Standard error of measurement
SHC1: Src homology 2 domain-containing transforming protein 1
StAR: Steroidogenic acute regulatory protein
TBP: TATA-binding protein
TCEP: Tris(2-carboxyethyl)phosphine
TEAB: Triethylammonium bicarbonate buffer
Thc: Theca cells
TIMP3: Tissue inhibitor of metalloproteinases 3
VEGF: Vascular endothelial growth factor
VEGFR2: Vascular endothelial growth factor receptor 2
VIS: Visfatin
