# Supplementary material for: Visfatin impact on the proteome of porcine luteal cells during implantation
Source: Sci Rep. 2024 Jun 25;14:14625. doi: 10.1038/s41598-024-65577-1 (PMC11199572; doi:10.1038/s41598-024-65577-1)

C - control group

VIS - visfatin-treated group

M – protein ladder (marker)

ADAMTS1 (predicted molecular weight 61 kDa)

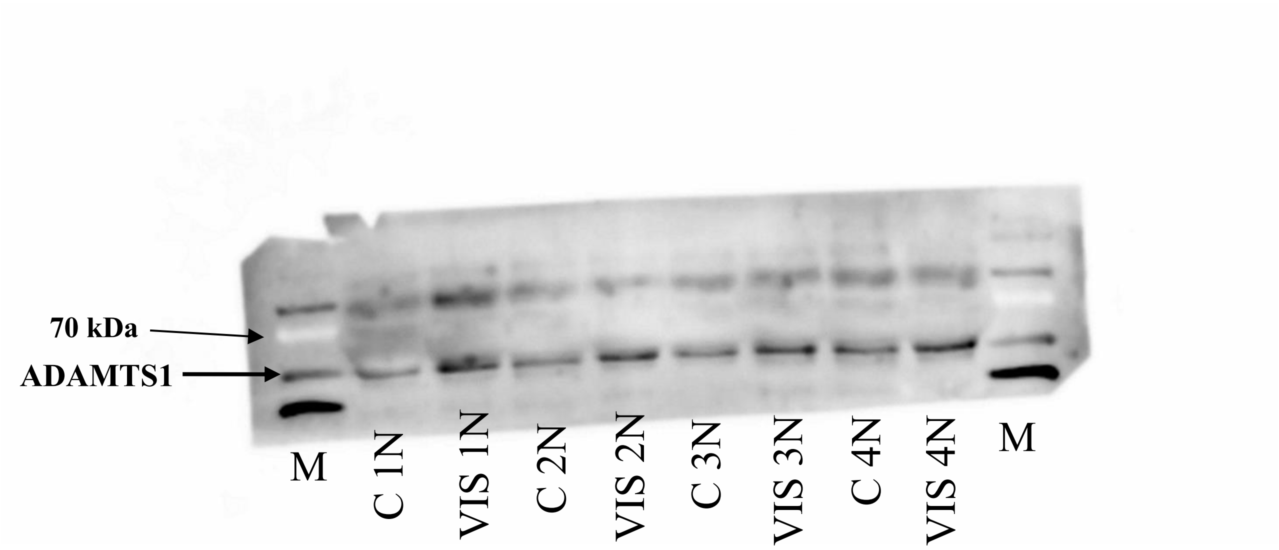

CYP51A1 (predicted molecular weight 55 kDa)

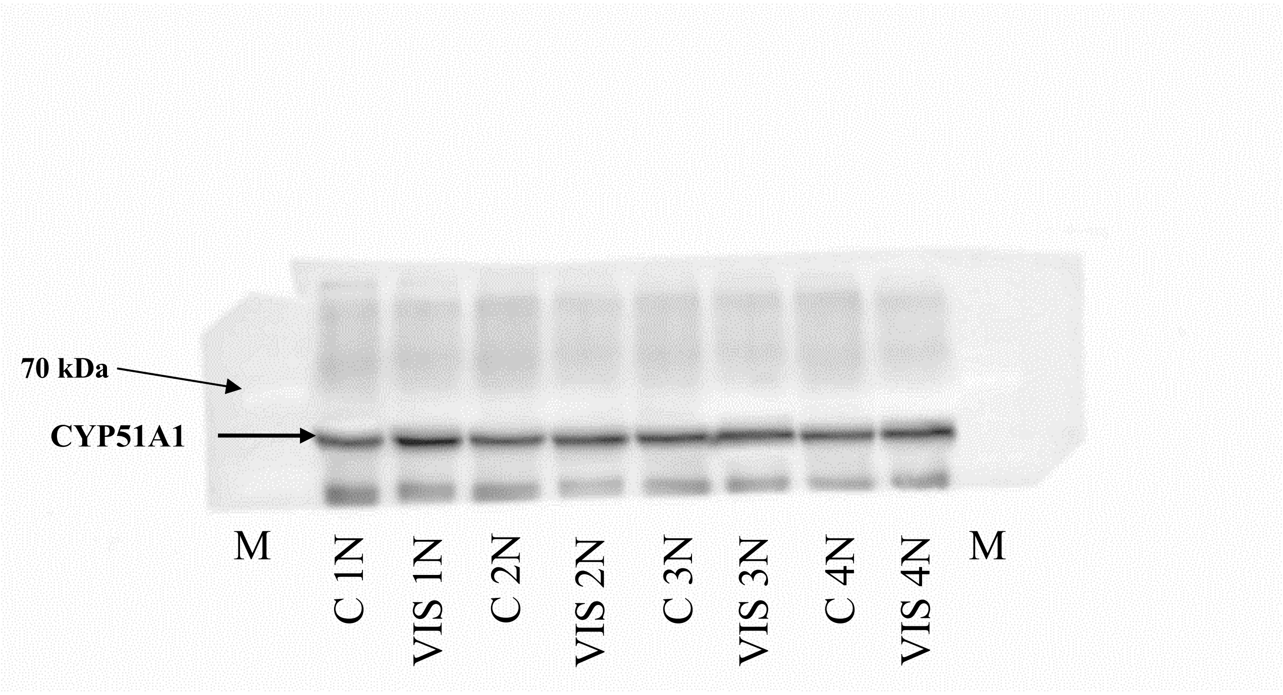

NOTCH3 (predicted molecular weight 90 kDa)

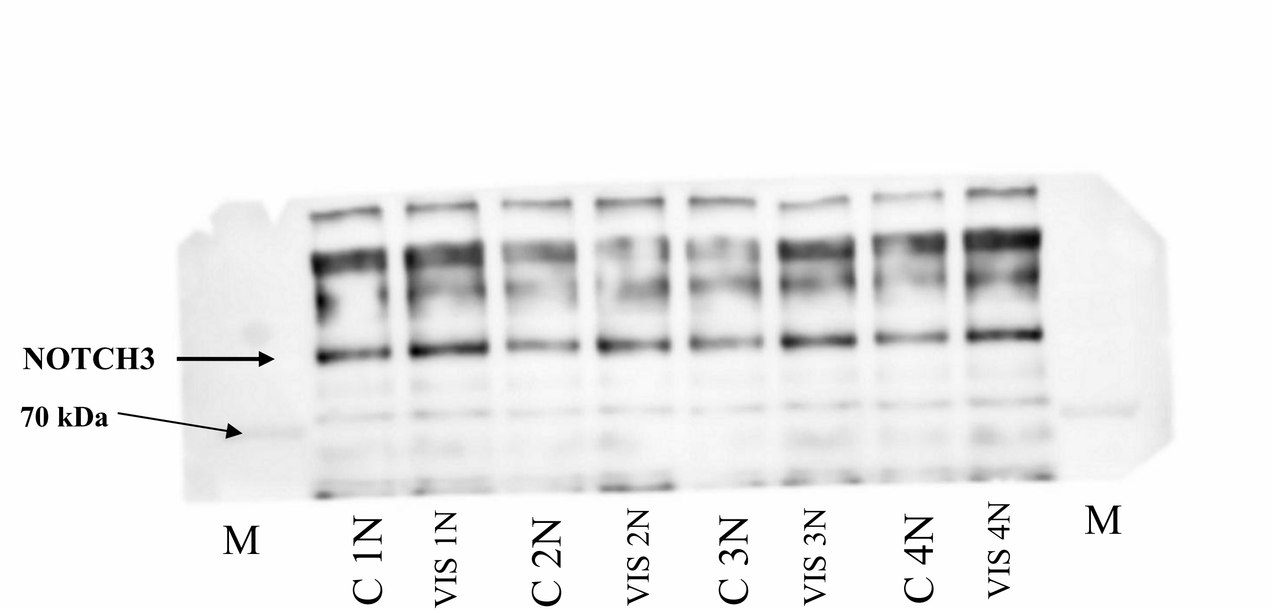

INHBA (predicted molecular weight 47 kDa)

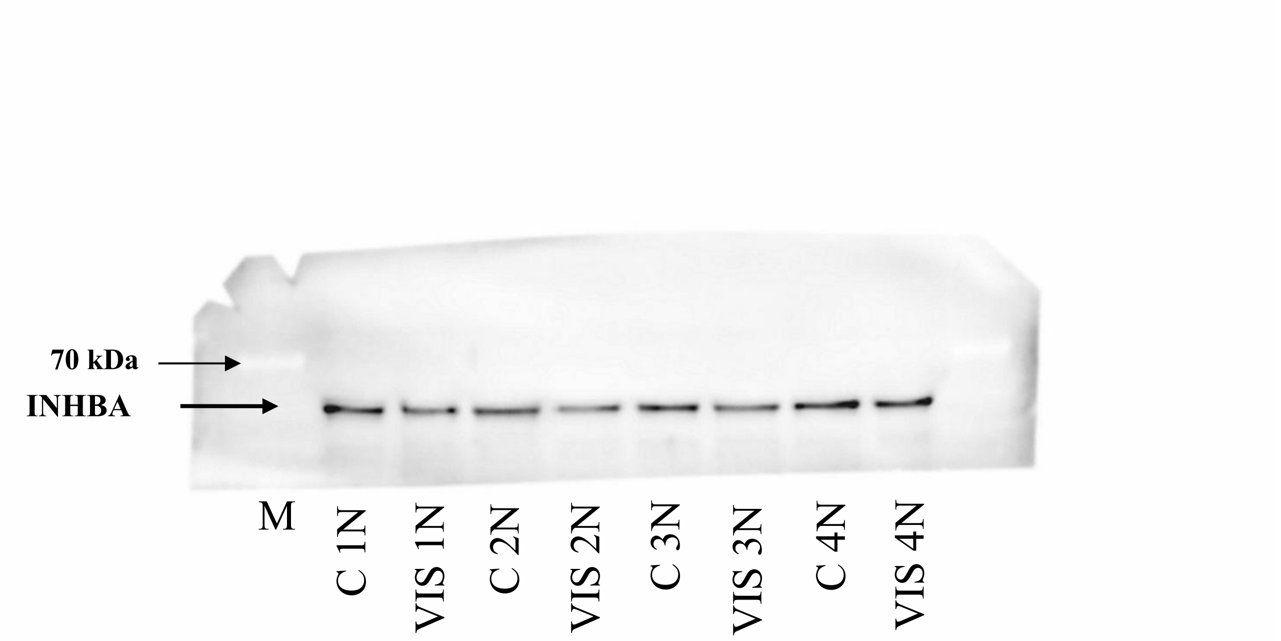

mPGES2 (predicted molecular weight 37 kDa)

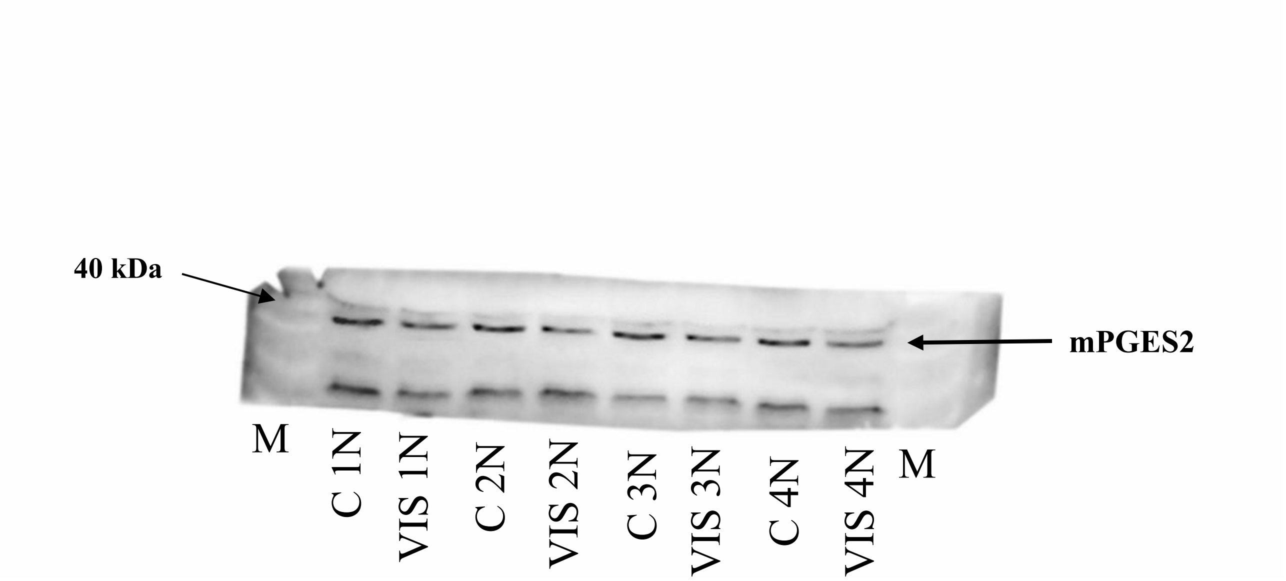

TBP (predicted molecular weight 38 kDa, reference protein)

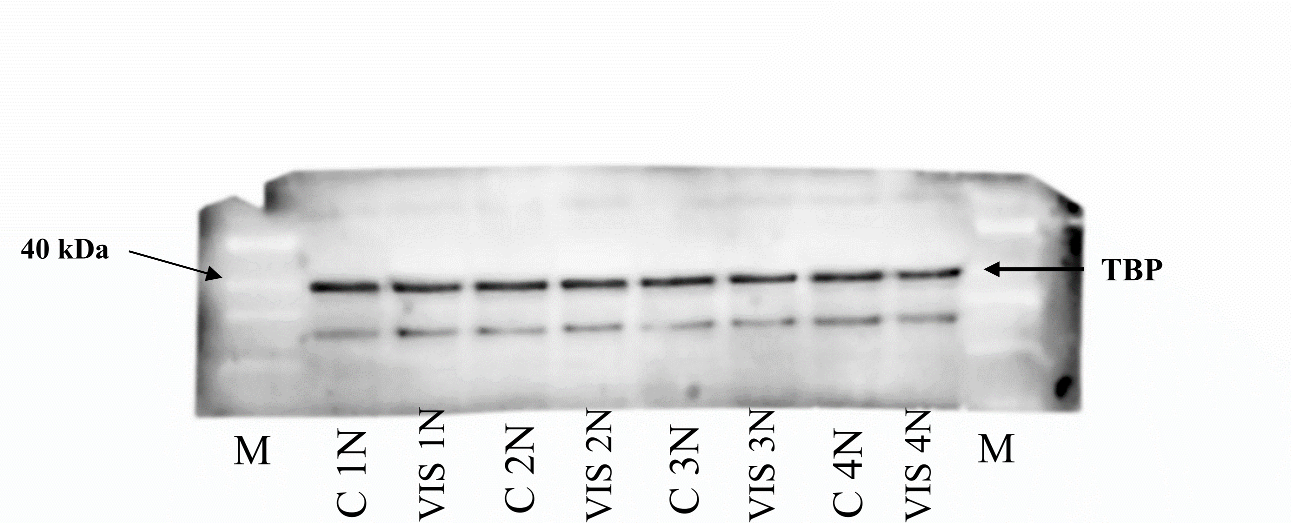

Supplement: Supplementary file 1 — Supplementary Information. [file 41598_2024_65577_MOESM1_ESM.pdf]
