# Supplementary material for: Visfatin impact on the proteome of porcine luteal cells during implantation
Source: Sci Rep. 2024 Jun 25;14:14625. doi: 10.1038/s41598-024-65577-1 (PMC11199572; doi:10.1038/s41598-024-65577-1)
Supplement: Supplementary file 2 — Supplementary Legends. [file 41598_2024_65577_MOESM2_ESM.pdf]

Supplementary Information

Supplementary information accompanies this paper.

Supplementary Table 1. Total proteome changes in the porcine luteal cells induced by visfatin treatment;

Supplementary Table 2. The differentially regulated proteins in luteal cells after visfatin treatment;

Supplementary Table 3. Gene Ontology and Kyoto Encyclopedia of Genes and Genomes signalling pathways enrichment analysis results.

Supplementary File 1. Uncropped images of immunoblots.

Supplementary File 2. Supplementary Files and Tables Legends
